# Income Inequality, Household Income, and Mass Shooting in the United States

**DOI:** 10.3389/fpubh.2018.00294

**Published:** 2018-10-17

**Authors:** Joseph F. Cabrera, Roy Kwon

**Affiliations:** Sociology-Anthropology Department, University of La Verne, La Verne, CA, United States

**Keywords:** mass shootings, income inequality, household income, relative deprivation theory, crime and criminal behavior

## Abstract

Mass shootings are becoming a more common occurrence in the United States. Data show that mass shootings increased steadily over the past nearly 50 years. Crucial is that the wide-ranging adverse effects of mass shootings generate negative mental health outcomes on millions of Americans, including fear, anxiety, and ailments related to such afflictions. This study extends previous research that finds a strong positive relationship between income inequality and mass shootings by examining the effect of household income as well as the interaction between inequality and income. To conduct our analyses, we compile a panel dataset with information across 3,144 counties during the years 1990 to 2015. Mass shootings was measured using a broad definition of three or more victim injuries. Income inequality was calculated using the post-tax version of the Gini coefficient. Our results suggest that while inequality and income alone are both predictors of mass shootings, their impacts on mass shootings are stronger when combined via interaction. Specifically, the results indicate areas with the highest number of mass shootings are those that combine both high levels of inequality and high levels of income. Additionally, robustness checks incorporating various measures of mass shootings and alternative regression techniques had analogous results. Our findings suggest that to address the mass shootings epidemic at its core, it is essential to understand how to stem rising income inequality and the unstable environments that we argue are created by such inequality.

## Introduction

The dramatic increase of mass shootings in the United States over the past few decades is an important topic of investigation for the field of public health. Although they are still relatively rare events compared to other forms of violence, mass shootings tend to spread fear and anxiety in the general population in a manner that extends well-beyond their specific geographic origins (1). Previous research finds a strong positive relationship between income inequality and mass shootings (2). Unexpectedly, this previous work also finds there is no connection between poverty and mass shootings. In this study, we extend on this prior work to test whether counties with higher income are more likely to experience mass shootings when compared to counties with lower income. Furthermore, we contend counties which simultaneously maintain high levels of income and inequality are the most likely to experience these events. We posit this potential interaction effect based on the premise that higher income communities may produce higher levels of frustration and anger resulting from an environment of extreme relative deprivation.

Anomie theory is one of the most well-known explanations connecting income inequality to violence and crime. According to Merton (3), those living in communities with sizable disparities in income experience high levels of relative deprivation, creating an environment which is rife with anger, frustration, resentment, and hostility. Often referred to as goal blockage, the effects of social strain can be particularly severe when a population finds it difficult to achieve socioeconomic success and status, leading to higher rates of crime and violence (4). We borrow from strain theory and suggest that inequality, especially in conjunction with high incomes, leads to unstable environments. Furthermore, such environments create a situation in which violent events such as mass shootings are more likely to occur.

In terms of income inequality, recent work supports such strain-based theories showing that inequality can lead to aggression. In one study, researchers found economy passengers who walked through the first class cabin were more than twice as likely to have an air rage incident than economy passengers who did not (5). A second study found that unequal environments are correlated with acts of aggression in the form of school bullying (6). These findings support the notion that inequality, and the salience of class and status differences it creates, can lead to unstable and hostile environments. While this is an example of situational inequality, we argue that structural inequality at the county level produces an environment in which situational occurrences of anger and aggression can occur more frequently, and may have cumulative effects on a community over time.

While there are no studies that specifically focus on how an inequality-income interaction is connected to mass shootings, some studies do suggest wealthy populations are more likely to partake in morally suspect behavior in communities with higher rather than lower levels of inequality. These studies find wealthier subsections of highly unequal populations view themselves as superior to others (7), believe they are more deserving (8), believe resources of the community rightly belong to them (9), and are less willing to share resources they view as scarce (10). As such, some researchers suggest inequality produces a “hitherto undiscovered effect of economic inequality on the psychology and behavior of high-income individuals” in populations characterized by both high levels of inequality and high levels of income [(9): p.15838]. In this way, it is not hard to imagine why the sorts of anger, frustration, and resentment theorized by the relative deprivation perspective would be pervasive in high inequality-income environments. In what follows, not only do the results of this study demonstrate mass shootings should be studied in relation to inequality, but the evidence also indicates there is an inequality-income interaction.

## Materials and methods

To conduct our analyses, we compile a panel dataset with information across 3,144 counties during the years 1990 to 2015. The dependent variable is from the Mass Shootings in America (MSA) dataset available at https://library.stanford.edu/projects/mass-shootings-america. All independent variables discussed below are from the U.S. Bureau of the Census (1990, 2000, 2010) available online through http://www.census.gov/data.html. Pooled panel regressions are the principal models in our study. In these models, mass shootings over a 10 year-period are regressed on independent variables measured during the years 1990, 2000, and 2010, respectively (e.g., mass shooting_1990−1999_ = inequality_1990_ + income_1990_ + ${\text{inequality}}_{1990}^{*}$income_1990_ + controls_1990_). Mass shootings was measured using the broader definition of three or more victim injuries as opposed to the more restrictive definition of four or more victim deaths. However, all models were analyzed with the more restrictive definition of mass shootings and results were nearly identical. We thus only present results for the broader definition. Additionally, we follow previous empirical studies and exclude shootings that are gang- or drug-related (11). There are two key independent variables in this study. First, income inequality is calculated using the post-tax version of the Gini coefficient, a measure which varies between 0 and 100, with higher scores denoting greater levels of income inequality. Second, median household income is used to measure county-level income. These data are inflation adjusted and reported in 2010 dollars. All models are calculated using STATA 13.0. A table of descriptive statistics can be found in (Table 1 in Appendix).

## Results

The findings of the negative binomial regression model (Table 1, Model 1), which examines the main predictors net of several control variables, indicates both income inequality (Adjusted IRR 1.46; 95% CI = 1.25, 1.71) and household income (Adjusted IRR 1.53; 95% CI = 1.26, 1.87) produce a statistically significant relationship with incidences of mass shootings. These results suggest that we should see a 0.46 increase in the expected number of mass shootings for every one standard deviation increase in inequality and a 0.53 increase for household income. In terms of the controls: population density, young population, and minority population return significant positive associations. Conversely, unemployment rate fails to return a significant relationship.

**Table 1:** Incidence rate ratios of mass shootings in U.S. counties.

	**Main Models** = **Panel Negative Binomial**	
	**Model 1 Adj. IRR (95% CI)**	**Model 2 Adj. IRR (95% CI)**
Income inequality	1.46^***^ (1.25, 1.71)	0.46^*^ (0.23, 0.89)
Household income	1.53 ^***^ (1.26, 1.87)	0.50^*^ (0.27, 0.94)
Inequality x income		3.01 ^***^ (1.62, 5.60)
Unemployment rate	1.17 (0.96, 1.42)	1.17 (0.96, 1.43)
Population density	2.10 ^***^ (1.70, 2.58)	2.04 ^***^ (1.66, 2.53)
Young population	1.24 ^***^ (1.10, 2.58)	1.26 ^***^ (1.11, 1.42)
Minority population	1.22^*^ (1.03, 1.46)	1.25^*^ (1.04, 1.51)
2000-2009	0.96 (0.63, 1.47)	0.99 (0.64, 1.52)
2010-2015	2.37 ^***^ (1.69, 3.33)	3.04 ^***^ (2.08, 4.44)
Chi-Square	456.68	440.24
County-Decades (N)	9415	9415
Years	1990–2015	1990–2015

* p < 0.05;

** p < 0.01;

*** *p < 0.001; Adj*.

*IRR, adjusted incidence rate ratio; CI, confidence interval*.

*All independent variables are logged and z-score standardized; Adjusted models are estimated by controlling for all independent variables; Robust clustered standard errors reported; Model fit statistics reported only for adjusted models*.

*Index of variables: income inequality = Gini coefficient that ranges 0–100 with higher scores denoting more inequality; household income = household income in 2010 U.S. dollars; unemployment rate = percent of the population without work and seeking employment; population density = individuals living in a county per square mile; young population = percent of the population aged 15 to 29; minority population = percent of the population that is non-White; HS graduation rate = percent of the population over the age of 25 with a high school or equivalent degree*.

**Figure 1:**
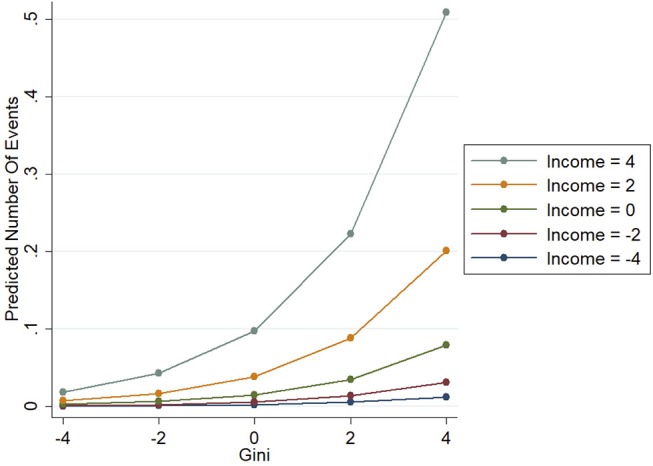
Linear prediction of mass shootings by various levels of household income and income inequality. Variables reported as standard deviations.

Results of the second model (Table 1, Model 2) show the interaction between inequality and income is positively associated with mass shootings (Adjusted IRR 3.01; 95% CI = 1.62, 5.60). In contrast to Model 1, both the main effects of inequality (Adjusted IRR 0.46; 95% CI = 0.23, 0.89) and income (Adjusted IRR 0.50; 95% CI = 0.27, 0.94) return non-significant negative associations with mass shootings. This result suggests that most of the variation in mass shooting is due to the interaction effect of inequality and income rather than any individual influence of either variable alone. In terms of the controls, we see the same patterns as in the first model. To make the interpretation of the interaction more comprehensible we include a figure (Figure 1) plotting the predicted number of mass shooting events with varying levels of inequality and household income^1^. Overall, the figure illustrates a key outcome, that inequality has a much stronger association with mass shootings in high-income counties than low-income counties.

## Discussion

This research suggests that understanding the connection between inequality and mass shootings, especially in high-income communities, could be instrumental in reducing the likelihood of this very real public health threat. Our results support previous work on the relationship between inequality and aggression (5, 6) and extend work on mass shootings by examining the role of household income (2). Specifically, the key contribution of this research is the finding of a strong interaction effect between inequality and income on mass shootings. That is, our results suggest inequality and income alone cannot adequately explain mass shootings. However, when their effects are combined, the interaction is strongly associated with the incidence rate of mass shootings. Given these results, we contend counties that are the most susceptible to mass shootings are those with both high levels of inequality and income.

One limitation of the MSA dataset used in this analysis is that sources are drawn from media reports. This means mass shootings that predate the Internet are less likely to be reported. Furthermore, incidents with sparse media attention are less likely to be included. To address these concerns we re-estimate all regression models by decade (Appendix, table 2). These results show little variation between income inequality and mass shootings over time, a result that is most robust during later years when data should be least biased, suggesting that our results are not an artifact of the data source. Previous studies also examined similar models using two other mass shootings datasets (USA Today and Mother Jones), showing outcomes did not vary by dataset (2). Based on our diagnostics, we are confident the correct method (negative binomial regression) was used in our analyses. However, we also run these models using other statistical techniques (zero-inflated, Poisson, multilevel models) to ensure our outcomes are not a side-effect of the analytical method used. All alternative models show similar statistically significant outcomes as our main model (Appendix, Table 3).

In closing, we posit that the link between the inequality-income interaction and mass shootings can be understood through the relative deprivation perspective, which contends the persistent inability of members of a community to achieve a culturally defined level of economic success creates an environment of anger, frustration, hostility, and violence. While we feel confident that relative deprivation and associated perspectives can shed light on mechanisms underlying mass shootings, much more work is needed to further support our claims. Nevertheless, what our results suggest is that communities most at-risk for mass shootings include the top economic engines of the United States such as New York and San Francisco, both of which maintain high levels of income inequality and household income. This may imply that to address the mass shootings epidemic at the population-level, it is essential to understand how economies can create economic success while also minimizing the staggering inequality, and correspondingly volatile environments, that often accompany such success.

## Author contributions

Both authors contributed equally to this work, each making a substantial, direct and intellectual contribution, and approved it for publication.

### Conflict of interest statement

The authors declare that the research was conducted in the absence of any commercial or financial relationships that could be construed as a potential conflict of interest.

## Footnotes

^1^Note that Figure 1 has been constructed using aggregated, rather than pooled data, as this technique makes the outcomes more interpretable, while not meaningfully changing the result.

## References

[1] Winett L Constructing violence as a public health problem. Public Health Rep. (1998) 133:498–507. PMC1308432 9847921

[2] Kwon R Cabrera JF Socioeconomic factors and mass shootings in the united states. Crit. Public Health. (Forthcoming). 10.1186/s12889-019-7490-x PMC6753615 31537201

[3] Merton RK Social Theory and Social Structure. New York, NY: Free Press (1968).

[4] Dollard J Miller NE Doob LW Mowrer OH Sears RR Frustration and Aggression. New Haven, NJ: Yale University Press (1939).

[5] DeCelles KA Norton MI. Physical and situational inequality on airplanes predicts air rage. Proc Natl Acad Sci USA. (2016) 113:5588–91. 10.1073/pnas.1521727113 27140642 PMC4878482

[6] Elgar FJ Pickett KE Pickett W Craig W Molcho M Hurrelmann K. School bullying, homicide and income inequality: a cross-national pooled time series analysis. Int J Public Health (2013) 58:237–45. 10.1007/s00038-012-0380-y 22714137

[7] Newman BJ Johnston JD Lown PL False consciousness or class awareness? Local income inequality, personal economic position, and belief in Amercan meritocracy. Am J Polit Sci. (2015) 59:326–40. 10.1111/ajps.12153

[8] Cambell WK Bonacci AM Shelton J Exline JJ Bushman BJ Psychological entitlement: interpersonal consequences and validation of a self-report measure. J Pers Assess. (2004) 83:29–45. 10.1207/s15327752jpa8301_04 15271594

[9] Acs ZJ Why Philanthropy Matters: How the Wealthy Give, and What It Means for Our Economic Well-Being. Princeton, NJ: Princeton University Press (2013).

[10] Cote J House J Willer R. High economic inequality leads higher-income individuals to be less generous. Proc Natl Acad Sci. USA. (2015) 112:15838–43. 10.1073/pnas.1511536112 26598668 PMC4702979

[11] Duwe G Kovandzic T Moody CE The impact of right-to-carry concealed firearm laws on mass public shootings. Homicide Stud. (2002) 6:271–96. 10.1177/108876702237341

