# Supplementary material for: Income Inequality, Household Income, and Mass Shooting in the United States
Source: Front Public Health. 2018 Oct 17;6:294. doi: 10.3389/fpubh.2018.00294 (PMC6199901; doi:10.3389/fpubh.2018.00294)
Supplement: Supplementary file 1 [file Table_1.DOCX]

**Appendix**

| **Table 1.** Descriptive Statistics  ****  *The Number of Mass Shootings variable is a count of mass shootings that occurred over each decade, all other variables are measured as of the census date at beginning of the time period. For Number of Mass Shootings, parenthesis in the range column indicates the total number of mass shootings for that time period.*  *Index of variables:* income inequality = Gini coefficient that ranges 0-100 with higher scores denoting more inequality; household income = household income in 2010 U.S. dollars; unemployment rate = percent of the population without work and seeking employment; population density = individuals living in a county per square mile; young population = percent of the population aged 15 to 29; minority population = percent of the population that is non-White; HS graduation rate = percent of the population over the age of 25 with a high school or equivalent degree.  **Table 2.** Incidence Rate Ratios of Mass Shootings in U.S. Counties, By Decade | | | | | | | | | |  |  |  |
| --- | --- | --- | --- | --- | --- | --- | --- | --- | --- | --- | --- | --- |
|  | 1990-1999 | |  | 2000-2009 | |  | 2010-2015 | |  |  |  |  |
|  | Adj. IRR (95% CI) | Adj. IRR (95% CI) |  | Adj. IRR (95% CI) | Adj. IRR (95% CI) |  | Adj. IRR (95% CI) | Adj. IRR (95% CI) |  |  |  |  |
|  |  |  |  |  |  |  |  |  |  |  |  |  |
| Income inequality | 1.47** (1.12, 1.92) | 0.10* (0.01, 0.85) |  | 1.47* (1.05, 2.06) | 0.06 (0.00, 1.07) |  | 1.58*** (1.28, 1.95) | 0.52** (0.00, 0.36) |  |  |  |  |
| Household income | 2.26*** (1.67, 3.07) | 0.20 (0.03, 1.23) |  | 1.97** (1.29, 2.99) | 0.10 (0.00, 1.45) |  | 1.27 (0.97, 1.65) | 0.04** (0.00, 0.28) |  |  |  |  |
| Inequality x income |  | 8.71** (1.71, 44.34) |  |  | 13.94* (1.24, 156.41) |  |  | 37.69** (4.66, 304.68) |  |  |  |  |
| Unemployment rate | 1.19 (0.87, 1.63) | 1.16 (0.85, 1.60) |  | 1.29 (0.89, 1.85) | 1.28 (0.89, 1.82) |  | 1.19 (0.87, 1.62) | 1.16 (0.85, 1.58) |  |  |  |  |
| Population density | 1.47* (1.04, 2.09) | 1.51* (1.03, 2.22) |  | 2.24*** (1.53, 3.28) | 2.25*** (1.55, 3.29) |  | 2.42*** (1.77, 3.31) | 2.36*** (1.71, 3.26) |  |  |  |  |
| Young population | 1.42* (1.08, 1.86) | 1.50* (1.09, 2.07) |  | 1.46** (1.11, 1.90) | 1.50** (1.14, 1.98) |  | 1.10 (0.90, 1.35) | 1.14 (0.92, 1.40) |  |  |  |  |
| Minority population | 1.17 (0.82, 1.67) | 1.18 (0.77, 1.82) |  | 1.06 (0.77, 1.44) | 1.13 (0.80, 1.60) |  | 1.34* (1.05, 1.70) | 1.45** (1.12, 1.88) |  |  |  |  |
|  |  |  |  |  |  |  |  |  |  |  |  |  |
| Chi-Square | 139.29 | 127.07 |  | 73.84 | 81.29 |  | 146.04 | 156.78 |  |  |  |  |
| Counties (N) | 3135 | 3135 |  | 3139 | 3139 |  | 3141 | 3141 |  |  |  |  |
|  |  |  |  |  |  |  |  |  |  |  |  |  |
| *Legend:* *p<.05; **p<.01; ***p<.001; Adj. IRR = adjusted incidence rate ratio; CI = confidence interval.  *Notes:* All independent variables are logged and z-score standardized; Adjusted models are estimated by controlling for all independent variables; Robust clustered standard errors reported; Model fit statistics reported only for adjusted models.  *Index of variables:* income inequality = Gini coefficient that ranges 0-100 with higher scores denoting more inequality; household income = household income in 2010 U.S. dollars; unemployment rate = percent of the population without work and seeking employment; population density = individuals living in a county per square mile; young population = percent of the population aged 15 to 29; minority population = percent of the population that is non-White; HS graduation rate = percent of the population over the age of 25 with a high school or equivalent degree. | | | | | | | | |  |  |  |  |

| **Table 3.** Incidence Rate Ratios of Mass Shootings in U.S. Counties | | | | | |  |  |  |
| --- | --- | --- | --- | --- | --- | --- | --- | --- |
|  | Zero-Inflated Models | |  | Poisson Models | |  | Multilevel Models | |
|  | Adj. IRR (95% CI) | Adj. IRR (95% CI) |  | Adj. IRR (95% CI) | Adj. IRR (95% CI) |  | Adj. IRR (95% CI) | Adj. IRR (95% CI) |
|  |  |  |  |  |  |  |  |  |
|  |  |  |  |  |  |  | Level 1 = County | |
|  |  |  |  |  |  |  |  |  |
| Income inequality | 1.51*** (1.26, 1.81) | 0.34* (0.14, 0.80) |  | 1.37*** (1.15, 1.63) | 0.60 (0.30, 1.17) |  | 1.36** (1.09, 1.71) | 0.51 (0.19, 1.40) |
| Household income | 1.59*** (1.28, 1.96) | 0.38* (0.16, 0.86) |  | 1.50*** (1.23, 1.84) | 0.69 (0.35, 1.35) |  | 1.29* (1.05, 1.59) | 0.51 (0.21, 1.25) |
| Inequality x income |  | 4.13*** (1.80, 9.45) |  |  | 2.16* (1.11, 4.24) |  |  | 2.53* (1.00, 6.40) |
| Unemployment rate | 1.19 (0.97, 1.45) | 1.19 (0.98, 1.45) |  | 1.16 (0.94, 1.42) | 1.15 (0.94, 1.41) |  | 0.91 (0.69, 1.21) | 0.93 (0.69, 1.25) |
| Population density | 2.15*** (1.70, 2.73) | 2.10*** (1.65, 2.67) |  | 1.97*** (1.60, 2.43) | 1.92*** (1.55, 2.37) |  | 2.51*** (1.88, 3.37) | 2.48*** (1.85, 3.32) |
| Young population | 1.28** (1.10, 1.48) | 1.30*** (1.12, 1.51) |  | 1.22** (1.09, 1.38) | 1.23*** (1.09, 1.38) |  | 1.24*** (1.10, 1.39) | 1.25*** (1.11, 1.42) |
| Minority population | 1.22* (1.01, 1.47) | 1.25* (1.03, 1.52) |  | 1.25* (1.05, 1.50) | 1.29** (1.07, 1.55) |  | 1.33** (1.07, 1.65) | 1.34** (1.08, 1.67) |
| 2000-2009 | 0.96 (0.63, 1.49) | 0.98 (0.63, 1.54) |  | 0.98 (0.64, 1.50) | 1.01 (0.65, 1.57) |  | 0.94 (0.64, 1.37) | 0.95 (0.65, 1.39) |
| 2010-2015 | 2.37*** (1.67, 3.36) | 3.13*** (2.11, 4.66) |  | 2.40*** (1.70, 3.37) | 2.96*** (2.04, 4.29) |  | 2.40*** (1.64, 3.49) | 2.96*** (1.97, 4.43) |
|  |  |  |  |  |  |  |  |  |
|  |  |  |  |  |  |  | Level 2 = State | |
|  |  |  |  |  |  |  |  |  |
| Right to carry | --- | --- |  | --- | --- |  | 1.02 (0.38, 2.74) | 0.94 (0.34, 2.64) |
| Assault rife ban | --- | --- |  | --- | --- |  | 0.59 (0.17, 1.96) | 0.58 (0.15, 2.17) |
|  |  |  |  |  |  |  |  |  |
| Chi-Square | 318.87 | 291.69 |  | 395.79 | 357.11 |  | 206.14 | 191.51 |
| County-Decades (N) | 9415 | 9415 |  | 9415 | 9415 |  | 9415 | 9415 |
| Years | 1990-2015 | 1990-2015 |  | 1990-2015 | 1990-2015 |  | 1990-2015 | 1990-2015 |
|  |  |  |  |  |  |  |  |  |
| *Legend:* *p<.05; **p<.01; ***p<.001; Adj. IRR = adjusted incidence rate ratio; CI = confidence interval.  *Notes:* All independent variables are logged and z-score standardized; Adjusted models are estimated by controlling for all independent variables; Robust clustered standard errors reported; Model fit statistics reported only for adjusted models.  *Index of variables:* income inequality = Gini coefficient that ranges 0-100 with higher scores denoting more inequality; household income = household income in 2010 U.S. dollars; unemployment rate = percent of the population without work and seeking employment; population density = individuals living in a county per square mile; young population = percent of the population aged 15 to 29; minority population = percent of the population that is non-White; HS graduation rate = percent of the population over the age of 25 with a high school or equivalent degree. | | | | | | | | |
